# Gut Microbiota Contributes to the Growth of Fast-Growing Transgenic Common Carp (*Cyprinus carpio* L.)

**DOI:** 10.1371/journal.pone.0064577

**Published:** 2013-05-31

**Authors:** Xuemei Li, Qingyun Yan, Shouqi Xie, Wei Hu, Yuhe Yu, Zihua Hu

**Affiliations:** 1 Key Laboratory of Aquatic Biodiversity and Conservation of Chinese Academy of Sciences, Institute of Hydrobiology, Chinese Academy of Sciences, Wuhan, China; 2 State Key Laboratory of Freshwater Ecology and Biotechnology, Institute of Hydrobiology, Chinese Academy of Sciences, Wuhan, China; 3 Center for Computational Research, New York State Center of Excellence in Bioinformatics and Life Sciences, Department of Ophthalmology, Department of Biostatistics, Department of Medicine, State University of New York at Buffalo, Buffalo, New York, United States of America; 4 Key Laboratory of Freshwater Biodiversity Conservation, Ministry of Agriculture of China, Yangtze River Fisheries Research Institute, Chinese Academy of Fishery Sciences, Wuhan, China; International Atomic Energy Agency, Austria

## Abstract

Gut microbiota has shown tight and coordinated connection with various functions of its host such as metabolism, immunity, energy utilization, and health maintenance. To gain insight into whether gut microbes affect the metabolism of fish, we employed fast-growing transgenic common carp (*Cyprinus carpio* L.) to study the connections between its large body feature and gut microbes. Metagenome-based fingerprinting and high-throughput sequencing on bacterial 16S rRNA genes indicated that fish gut was dominated by Proteobacteria, Fusobacteria, Bacteroidetes and Firmicutes, which displayed significant differences between transgenic fish and wild-type controls. Analyses to study the association of gut microbes with the fish metabolism discovered three major phyla having significant relationships with the host metabolic factors. Biochemical and histological analyses indicated transgenic fish had increased carbohydrate but decreased lipid metabolisms. Additionally, transgenic fish has a significantly lower Bacteroidetes:Firmicutes ratio than that of wild-type controls, which is similar to mammals between obese and lean individuals. These findings suggest that gut microbiotas are associated with the growth of fast growing transgenic fish, and the relative abundance of Firmicutes over Bacteroidetes could be one of the factors contributing to its fast growth. Since the large body size of transgenic fish displays a proportional body growth, which is unlike obesity in human, the results together with the findings from others also suggest that the link between obesity and gut microbiota is likely more complex than a simple Bacteroidetes:Firmicutes ratio change.

## Introduction

Microbes, which colonize animal gut, function collectively as an extra ‘organ’ for the host. Their community structure is shaped by the combining effects of host genotype, lifestyle, living environments, and selective pressures from gut habitats [1]–[4]. The genomes of these microbes (microbiome) exceed the size of the host nuclear genome by a few orders of magnitude, contributing to a broad range of functions which have not evolved wholly on the host [5]–[7]. The genomes of the microbes, unlike its host genome, can dynamically change the configuration of their components to fulfill the needs of the community as a whole and of the host. A growing number of studies have shown gut microbiota has a tight and coordinated connection with host metabolism, energy utilization and storage, immunity and nutritional status, and health maintenance [8]–[11].

Obesity, which results from the accumulation of excess adipose tissue, presents a good example for illustrating the potential interactions between the mammalian host and its dynamic symbionts. Also, the dynamics of microbial genomic and metabolic diversity are key factors maintaining host’s health [8]. The causes driving obesity appear to be complex. A consensus hypothesis is a heterogeneous group of conditions with multiple causes, including behavioral and environmental factors such as a sedentary lifestyle and excessive consumption of energy-dense foods [12]. It has recently been proposed that gut microbiota, as an environmental factor, may shape the host immune network and metabolic activity which in turn alters energy metabolism accompanying the obese state. The potential mechanisms underlying this relationship include increased nutrient absorption from the diet, prolonged intestinal transit time, altered bile acid entero-hepatic cycle, increased cellular uptake of circulating triglycerides, and altered tissue composition of biologically active polyunsaturated fatty acid [13].

Although there have been about 30 bacterial phyla described to date, the development of obesity has most often been associated with significant changes to Bacteroidetes and Firmicutes levels. Obese mice resulting from a high-fat/high-sugar western diet, as compared with mice receiving a low-fat/high-polysaccharide diet, display enrichment in Firmicutes at the expense of Bacteroidetes [14]. Similar to animal models, obese people have a relatively higher proportion of Firmicutes, when compared to lean people. Surgically- or diet-induced weight loss can reduce the proportion of Firmicutes [15]–[18]. However, this finding is inconsistent from other studies. Duncan et al. have showed proportions of Bacteroidetes and Firmicutes among fecal bacteria have no association in human obesity [19]. In another study, overweight and obese subjects have a ratio of Bacteroidetes to Firmicutes in favor of Bacteroidetes [20]. Recently, Jumpertz et al. investigated dynamic changes of gut microbiota by applying pyrosequencing to examine bacterial 16S rRNA genes and reported no phylum level difference between fecal microbiota of obese and lean subjects [21]. Therefore, the link between obesity and the microbiota is likely more sophisticated than the simple phylum-level Bacteroidetes:Firmicutes ratio change.

In this report we conducted a series studies to reveal the relationships between gut microbes and the fast-growing feature of transgenic common carp, which was modified with an ‘all-fish’ growth hormone gene [22], [23]. Although the transgenic fish has a large body size stimulated by the recombinant grass carp (*Ctenopharyngodon idellus*) growth hormore gene (*gcGH*) when compared to wild-type controls, unlike obesity in human it displays an isometric body growth. In fact, significantly lower levels of growth hormone receptor (*GHR*) mRNA have been found in adipose tissues of obese human subjects, as compared with the lean human counterparts [24]. Furthermore, mice with growth hormone receptor deficiency (*GHR*−/−) have a greater percent fat mass but with no significant differences in absolute fat mass through the life, and animals with lean mass show an opposite trend [25]. It has been found that the organization of fish intestine is similar to that of mammals, and more importantly many homologous genes which are regulated by gut microbes in mammals show similar expression responses in fish [26]. Therefore the fast-growing transgenic fish provides a good model not only to study the impact of gut microbial communities on the growth of fish but also to investigate if the increase of Firmicutes at the expense of Bacteroidetes is unique to obesity.

By using metagenome-based methods, we found significant differences of gut microbiota composition between transgenic fish and wild-type controls during a two-year field study, while both displayed high degree of similarities within each group. The results were further confirmed by high-throughput sequencing on bacterial 16S rRNA genes. We further extended our study to reveal the association of gut microbes with fish metabolism and discovered three major phyla (Proteobacteria, Bacteroidetes, and Firmicutes) had significant relationships with the host metabolic factors. Furthermore, transgenic fish had increased carbohydrate but decreased lipid metabolisms, which were evidenced by both biochemical and histological analyses. Additionally, we observed that the fast-growing transgenic fish had a significantly lower Bacteroidetes:Firmicutes ratio than that of wild-type controls. The results indicate that the Bacteroidetes:Firmicutes ratio change is not unique to obesity. The results also suggest that the relative abundance of Firmicutes over Bacteroidetes could be one of the factors contributing to the fast growth of transgenic fish, although Bacteroidetes and Firmicutes account for only a small proportion of its gut microbiota.

## Materials and Methods

### Animals and Ethics Statement

Individuals of transgenic fish (*Cyprinus carpio* L.) at different developmental stages (from larvae to adults) were sampled, and counterparts from wild-type fish were used as controls. All experiments involving animals were performed under protocols approved by the Institutional Animal Care and Use Committee of Institute of Hydrobiology, Chinese Academy of Sciences (Approval ID: keshuizhuan 08529).

### Experimental Design and Fish Husbandry

Transgenic fish and wild-type controls were reared with the same commercial feed in ponds at Guanqiao Experimental Station. Individuals sampled from larval stage to adult animals (from April 2009 to March 2011) were used to study the structure and dynamics of gut microbiota. To study nutrient metabolism related to the gut microbiota, four different diets (Table S1) were given to both transgenic and wild-type fish (during the stages of 2-month and 5-month) under laboratory condition. Briefly, the transgenic fish and wild-type controls were transferred to the laboratory at the stage of 2-month, and then acclimated to laboratory condition with a practical diet twice a day (09∶00 AM and 16∶00 PM) for the first 3 weeks and an equal mixture of the four experimental diets (Table S1) for the 4^th^ week. At the beginning of the laboratory growth experiment, acclimated transgenic fish were weighed after one day of food deprivation and then randomly distributed into 12 tanks (20 individuals for each tank, totally about 60 g). Three tanks were randomly assigned as replicates for each dietary treatment. After a growth trial for six weeks, fish in each tank were also weighed after one day of food deprivation and then were randomly selected for analysis as described in the following sections.

### Intestine Sampling Procedures and Bacterial dna Preparation

For larval stages (3- and 6-day post-incubation), intestines were removed aseptically under a dissecting microscope, and three replicated samples for both transgenic fish and wild-type controls were used for investigating the diversity and dynamics of gut microbiota. For each of the late stages five (for individuals at the stages of 2–5 month) or three individuals (at the stages of 8–23 month) from both transgenic fish and wild-type controls were randomly selected and subject to the following procedures. The intestine was first carefully removed under sterile environments. Whole intestinal tract (for individuals at 2–5 month stages) or part of foregut, mid gut, and hind gut (for individuals at of 8–23 month stages) was then collected for subsequent DNA extraction. For fish reared in the laboratory condition three individuals from each diet treatment (one individual from each of triplicate tanks) were randomly selected and whole intestine of each individual were collected as described above.

DNA preparation was performed by incubating intestinal homogenates in 1 ml lysis solution (30 mM EDTA, 10 mMTris-HCl, 0.5% sodium dodecyl sulfate (SDS), 0.1 mg proteinase K, 0.05 mg RNase A) at 55°C bath for 10 h, followed by standard phenol/chloroform extraction and precipitating with cold ethanol as previously described [27].

### PCR-DGGE and Sequencing

To amplify bacterial 16S ribosomal RNA gene, PCR reactions (25 µL) were prepared, each containing approximately 1 ng/µl DNA templates, 1×buffer (without MgCl_2_), 2 mM MgCl_2_, 0.06 unit/µl*Taq* DNA polymerase, 80 µM of deoxynucleotide triphosphate, and 0.25 µM of each universal bacterial target primer 357F-GC and 518R [28] (Table S2). Touchdown PCRs were performed on a S1000™ thermal cycler (Bio-Rad) with the following conditions: 5 min at 94°C, followed by 10 cycles of 30 sec at 94°C, 30 sec at 66–57°C, and 60 sec at 72°C. This procedure was followed by 20 cycles of 30 sec at 94°C, 30 sec at 56°C and 60 sec at 72°C with a post-amplification extension of 10 min at 72°C. All PCR products were confirmed by agarose gel electrophoresis.

Approximately equal amounts of PCR products were separated by denaturing gradient gel electrophoresis (DGGE) using 9.0% polyacrylamide gel with a 45–70% denaturing gradient. Electrophoresis was performed at 60°C with 100 V for 12 h according to the method described previously [29]. Gels were then stained in 1×TAE buffer containing 1×SYBR Gold (Molecular Probes) for 30 min, followed by photographing with a Gel Doc™ XR imaging system (Bio-Rad). DGGE band types were originally assigned and matched using the Quantity One® software (Bio-Rad, version 4.6.9), and the banding patterns were then manually checked.

Dominant bacterial operation taxonomic units (OTUs) were recovered from DGGE profiles by excising the bands with relatively high density and re-amplified using the same primer pairs without GC-clamp (357F and 518R). The resulting products were visualized using 1.8% agarose gels. Target 16S rRNA gene fragments were excised and purified using agarose gel DNA extraction kit (Axygen), cloned into pMD18-T vector (TaKaRa), and then transformed *Escherichia coli* (DH-5a) with a plasmid. Two positive clones for each OTU were sequenced.

All partial 16S rRNA gene sequences were compared with those in the public Ribosomal Database Project II [30] to ascertain their closest relatives. Neighbor-joining phylogenetic trees were calculated using ClustalX in combination with MEGA (4.0) package [31]. Bootstrap (1000) was performed to evaluate the phylogenetic tree.

### Quantifying Firmicutes and Bacteroidetes by Q-PCR

Real-time quantitative PCR (Q-PCR) was used to quantify the relative abundance of gut Firmicutes and Bacteroidetes [32] using standards constructed with known amounts of plasmid DNA. In brief, PCR products of 16S rRNA genes were gel-purified, cloned into pMD18-T vector, and then transformed into *Escherichia coli* cells. After confirming by sequencing, plasmid DNA containing cloned 16S rRNA gene was extracted. The resulting DNA concentrations were determined by spectrophotometry with serial dilutions. Standard curves were then established using diluted plasmid DNA in Q-PCR. The abundance of Firmicutes, Bacteroidetes, and total bacteria in each intestinal sample was evaluated.

The Q-PCR was performed on an ABI 7500 FAST system (Applied biosystems). Each PCR (25 µL) contains 1×SYBR Green qPCR master mix (Shanghai Ruian), 0.2 µM of each primer (Table S2), and 2 µL DNA templates. PCR cycling included an initial denaturation for 2 min at 95°C, followed by 40 cycles of 94°C for 10 sec, 60°C for 40 sec. Fluorescence readings were taken at each extension step, and a final melting analysis was performed to check nonspecific product formation. Three replicates were analyzed for each sample.

### Bacterial 16S rRNA Gene Pyrosequencing

The V1-V3 regions, which have more related variations for 16S rRNA gene than shorter sequences or the full-length sequence [33], were amplified using the bacterial primers 27F and 534R (Table S2) with Pyrobest™ DNA polymerase (Takara). The sample-unique 10-base bar-code was add to each primer for sorting of PCR amplicons into different samples, and the underlined text indicates universal bacterial primers. PCR products were purified with the QIAquick Gel Extraction Kit (Qiagen), after quantifying by the Qubit™ Quantitation Platform (Invitrogen), 200 ng product from each sample was pooled for pyrosequencing by a 454 GS FLX Titanium system (454 Life Sciences/Roche Applied Science) according to the manufacturer’s instructions. After pyrosequencing all reads were scored for quality filtering and the sequences that passed quality control were used to pick operational taxonomic units (OTUs). The representative sequence of each OTU was used for taxonomy assignment and generating phylogenetic tree. Alpha- and beta-diversity were also calculated for comparing bacterial communities, clustering and PCA were also performed to visually depict the differences between samples. All these analyses were performed according to the procedures described elsewhere [34]. The pyrosequencing dataset was deposited into European Nucleotide Archive under the accession number ERP002333.

### Statistical Analyses

Statistical analyses were performed with the software SPSS, R package, and XLSTAT. The pyrosequencing results were analyzed using the pipeline of QIIME [35] and the Fast UniFrac online toolkit (http://bmf2.colorado.edu/fastunifrac/) [36]. A binary matrix from DGGE band matching data was used to calculate Sørensen similarities for unweighted pair-group method with arithmetic average (UPGMA) clustering. The band patterns were also analyzed using the Raup and Crick probability-based index of similarity (*S*_RC_), which provides a measurement of statistically significant similarity and dissimilarity at the 95% confidence level [37]. The similarity index is the probability that the randomized similarity would be less than or equal to the observed similarity, and *S*_RC_ values above 0.95 or below 0.05 signifies the similarity or differences [38]. The *S*_RC_ was calculated using the PAST program. In addition, canonical ordination of redundancy analysis (RDA) was performed with Canoco for Windows 4.5 to screen microbial phyla that could significantly predict metabolic characters and to explore the potential relationships between intestinal microbes and host’s metabolism. One way ANOVA and two-tailed Student’s *t*-test were performed to assess the differences between transgenic fish and wild-type controls. For the time-series and multiple diet treatments the statistical significance between transgenic and wild-type fish was evaluated using one-side Wilcoxon signed-rank test and one-side paired *t*-test.

## Results

### Gut Microbiota Composition Differs between Transgenic Fish (*Cyprinus carpio* L.) And Wild-type Controls

**Figure 1:**
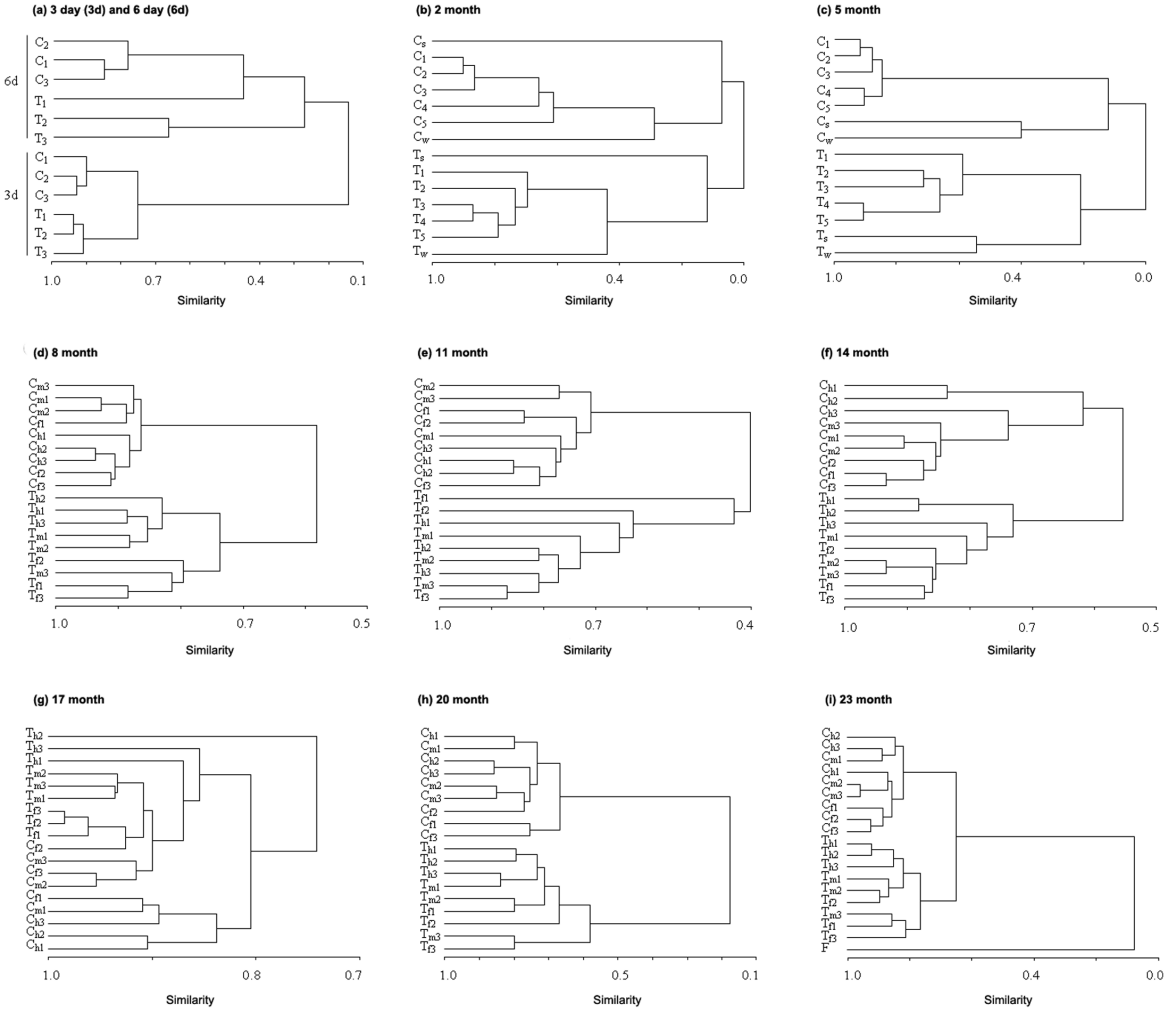
UPGMA clustering over Sørensen similarity of gut microbiota composition between transgenic fish and wild-type controls across different developmental stages. The similarity matrix was calculated using the binary data, and clustering was performed using the unweighted pair-group method with arithmetic average (UPGMA). *T_i_* and *C_i_* indicate the *i^th^* replication of transgenic fish and wild-type control, respectively; *T_fi_*, *T_mi_*, and *T_hi_* represent the *i^th^* foregut, midgut, and hindgut samples from transgenic fish, respectively, and *C_fi_*, *C_mi_*, and *C_hi_* from the corresponding part of controls; *T_s_* and *T_w_* represent the sediment and water samples collected from the pond where transgenic fish were reared, and *C_s_* and *C_w_* represent controls; *F* indicates food sample.

**Figure 2:**
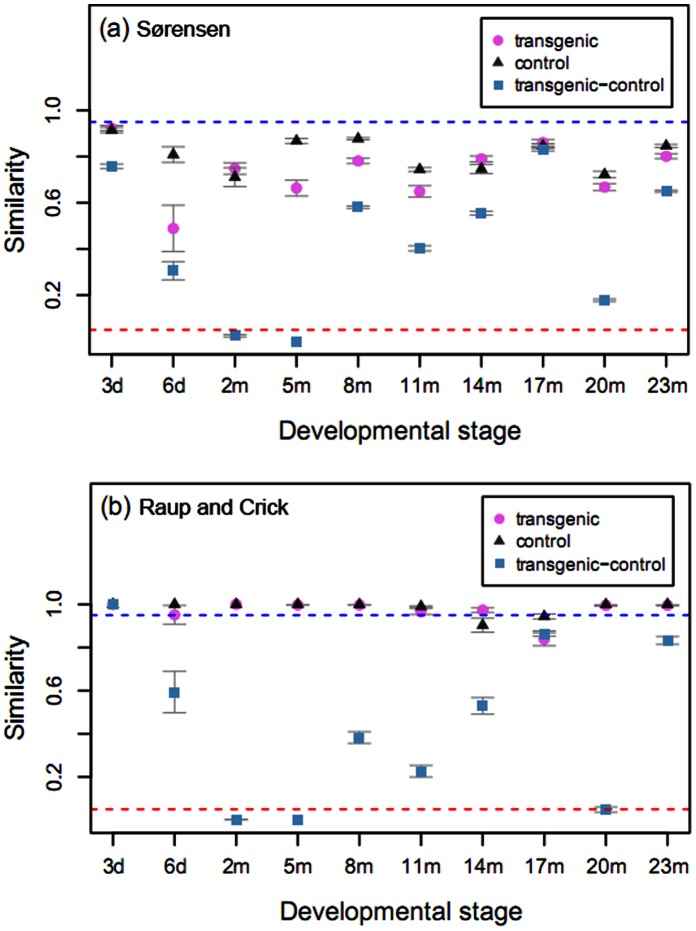
Comparison of similarities and differences for gut microbiota composition between transgenic fish and wild-type controls across different developmental stages. (a) Comparison of the average Sørensen index obtained from DGGE patterns of 16S rRNA genes between transgenic fish and wild-type controls or within each group of transgenic fish and wild-type controls. (b) Comparison of Raup and Crick similarity index (*S*_RC_) obtained from DGGE patterns of 16S rRNA genes between transgenic fish and wild-type controls or within each group of transgenic fish and wild-type controls. Dashed lines indicate significant cutoff for difference (low line) and similarity (upper line). Error bars represent the standard error of the mean.

A two-year field study was performed to explore the similarities and differences in gut microbiota composition between transgenic fish (represented by *T* in Figures and Tables) and wild-type controls (represented by *C* in Figures and Tables). Individual fish reared in ponds with the same commercial feed from larval stage to adult animals were used for the comparison of gut microbiota composition. Sørensen similarity based on DGGE patterns of 16S rRNA genes (V3 region) indicated that gut microbiotas in transgenic fish were different from those in wild-type controls as shown in Figure 1, where UPGMA clustering classifies samples of transgenic fish and wild-type controls into two distinct groups in all developmental stages from the field study (only the 17-month developmental stage showed some exception). Analyses were also performed to compare the mean value of Sørensen index either between transgenic fish and wild-type controls (between-group) or within each group of transgenic fish and wild-type controls (within-group). While both within-groups displayed high degree of similarities (0.81±0.07 and 0.74±0.12), the lowest similarities (0.43±0.30) was observed in the between-groups (Figure 2a). Statistical analyses using one-side Wilcoxon signed-rank test revealed significant differences (*p*<0.001) regarding Sørensen index between the within-groups and the between-group comparisons.

The degree of similarity was further assessed using Raup and Crick similarity index (*S*_RC_) [37], [38]. This probability-based similarity index tells whether the samples are significantly similar (*S*_RC_ ≥0.95), significantly dissimilar (*S*_RC_ ≤0.05), or have no significant difference (0.05< *S*_RC_ <0.95). Similar to the above findings *S*_RC_ was ≥0.95 for most within-group comparisons except the 17-month developmental stage for transgenic fish and the 14-month as well as 17-month stages for wild-type controls (Figure 2b), suggesting these within-group similarities were mainly driven by deterministic considerations. On the other hand, *S*_RC_ displayed low similarity between transgenic fish and wild-type controls with a mean value of 0.42±0.34. Significantly low *S*_RC_ (<0.05) was observed for the 2-, 5-, and 20-month developmental stages. Further statistical analyses using one-side Wilcoxon singed-rank test revealed significant differences (*p*<0.01) between the within-groups and the between-group. The *S*_RC_ values between environmental samples (sediment and water samples from the ponds) and corresponding gut samples ranged from 0.05 and 0.95 with a mean value of 0.42±0.30. These findings suggest that gut microbiota composition is not significantly similar to the environments.

**Figure 3:**
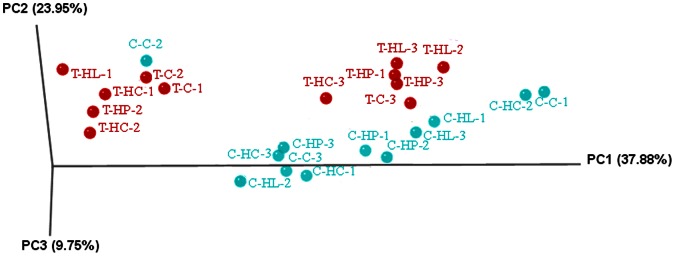
Principal Component Analysis (PCA plot with UniFrac scaled axis) of individual samples with different diet treatments. For each sample code, the first letter *T* represents transgenic fish, and *C* wild-type controls; the middle letter(s) indicates the diet treatments (*C*: control diet, *HP*: high protein diet, *HC*: high carbohydrate diet, *HL*: high lipid diet), and the last numbers represent replicate samples.

To confirm the findings from the DNA fingerprinting approaches and to further study gut microbe phylotypes, 454-pyrosequencing was applied to analyze 16S rRNA genes (V1-V3 regions). Gut samples collected from fish raised under laboratory condition with four different diets (in Figures: *C* represents control diet, *HP* represents high protein diet, *HC* represents high carbohydrate diet, and *HL* represents high lipid diet, Table S1) were used for this analysis. A total of 621,110 valid bacterial 16S rRNA gene reads were obtained. OTUs at 97% homology cutoff indicated the gut microbiota was dominated by Proteobacteria (59%–87%), Fusobacteria (6%–19%), Bacteroidetes (5%–16%), and Firmicutes (1%–3%). Principal component analysis (PCA plot with UniFrac scaled axis) indicated transgenic and control samples in general showed relatively higher similarities within each group than those of between groups (Figure 3). The difference of gut microbiota composition was also observed from the average OTU counts of the dominating bacterial phyla (Figure S1). The number of members unique to transgenic fish was between 18% (Proteobacteria) and 46% (Actinobacteria).

Alpha-diversity analysis of OTUs from different diet treatments revealed no significant differences (*p*>0.05) between wild-type controls and transgenic fish regarding the ACE, Chao1, and Shannon diversity, although wild-type controls had comparatively larger values than samples of transgenic fish except the high protein diet group (Table 1).

**Table 1:** Alpha-diversity of gut microbiota calculated according to the composition and relative abundance of OTUs with 97%-identity.

	Control diet treatment		High protein diet treatment		High carbohydrate diet treatment		High lipid diet treatment	
	T	C	T	C	T	C	T	C
Chao1	1522.31±236.06	2074.92±604.71	3355.92±790.68	2182.72±498.22	2116.61±44.06	2455.50±349.88	2814.63±443.53	2924.76±656.02
ACE	1597.77±244.93	2205.18±599.29	3408.50±800.13	2155.29±424.72	2108.88±53.83	2568.71±349.81	2888.23±481.03	3026.54±649.95
Shannon	5.83±0.32	6.41±0.69	6.26±0.68	5.82±0.44	5.59±0.45	6.52±0.67	5.67±0.88	6.18±0.31

### Transgenic Fish has a Low Bacteroidetes:Firmicutes Ratio

Previous studies indicated that obesity in humans and animals might be associated with decreased gut Bacteroidetes:Firmicutes ratio [14]–[18], [39], [40]. This characteristic has also been explored here to see whether it is unique to obesity.

**Figure 4:**
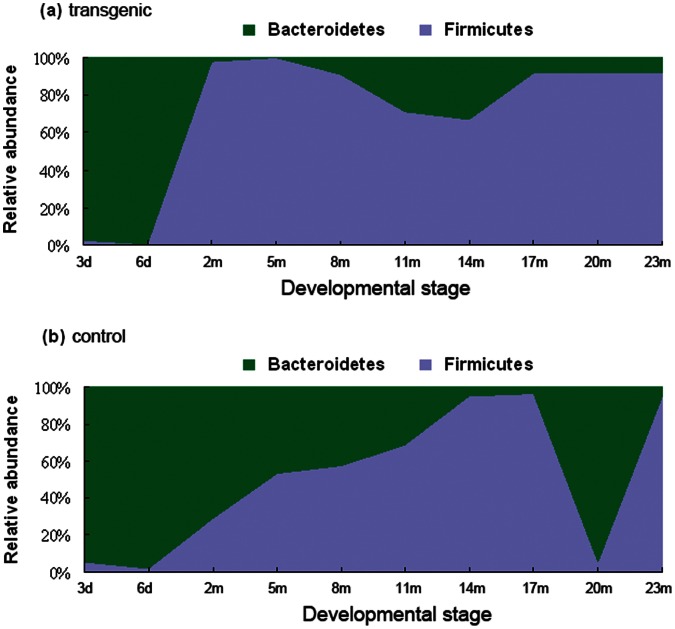
Comparison of relative Bacteroidetes and Firmicutes abundance at different developmental stages. Real-time quantitative PCR (Q-PCR) was used to quantify the abundance of gut Firmicutes and Bacteroidetes based on the 16S rRNA genes (V3 region). (a) Relative abundance of Firmicutes and Bacteroidetes in transgenic fish. (b) Relative abundance of Firmicutes and Bacteroidetes in wild-type controls.

Q-PCR quantification of 16S rRNA genes copies for these two bacterial phyla indicated that at the initial developmental stages (3- and 6-day) both transgenic fish (Figure 4a) and wild-type controls (Figure 4b) had very high Bacteroidetes:Firmicutes ratio. The relative Bacteroidetes abundance in transgenic fish decreased dramatically to less than 2% at the 2-month and the 5-month developmental stages. The trend of lower Bacteroidetes:Firmicutes ratio was retained through the rest of the late developmental stages, and the largest Bacteroidetes abundance only accounted for 33% (at the 14-month developmental stage). By contrast, wild-type fish displayed a relatively high Bacteroidetes:Firmicutes ratio. This was especially true at the developmental stages of 2-month, 5-month, and 20-month, for which the relative Bacteroidetes abundance was more than 71%, 46%, and 94%, respectively. One-side paired *t*-test indicated that the proportion of Bacteroidetes in wild-type controls was significantly larger than those in transgenic fish (*p* = 0.04). For fish raised in laboratory condition the relative abundance of Bacteroidetes and Firmicutes was detected by high-throughput sequencing based on 16S rRNA genes (V1-V3 regions). In agreement with the findings from fish raised in natural conditions, transgenic fish had a much lower Bacteroidetes:Firmicutes ratio than that of wild-type controls (data not shown).

The results indicate that the Bacteroidetes:Firmicutes ratio change is not unique to obesity. Although Bacteroidetes and Firmicutes account for only a small proportion of gut microbiota in fish, the finding from this study suggests that the relative abundance of Firmicutes over Bacteroidetes could be one of the factors contributing to the fast growth of transgenic common carp. The result together with the findings from others 19–21] also suggest that the link between obesity and gut microbiota is likely more complex than a simple change of Bacteroidetes:Firmicutes ratio.

### Proteobacteria, Bacteroidetes, and Firmicutes Display Significant Relationship with Metabolism of the Investigated Common Carp

**Figure 5:**
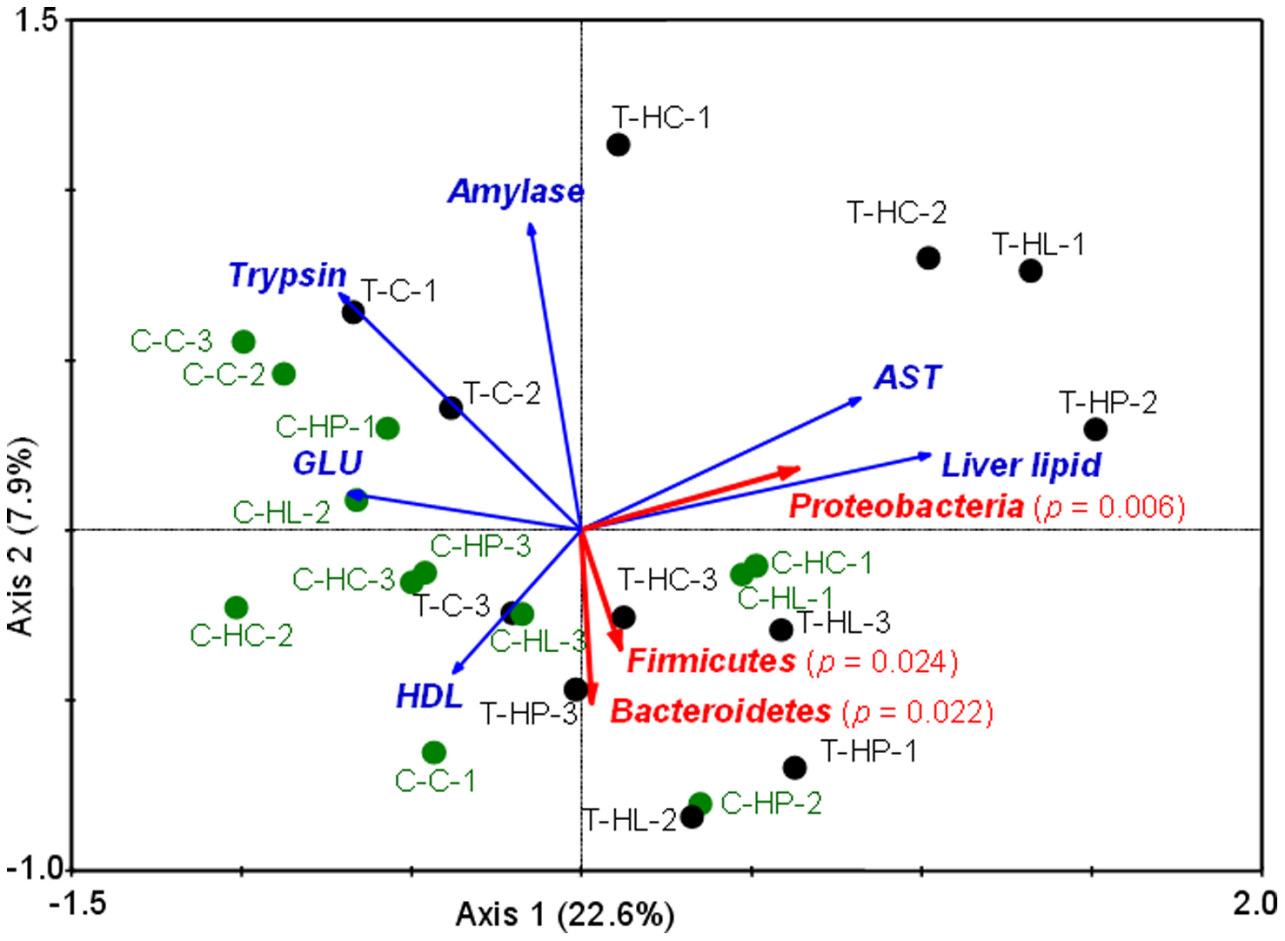
Triplot of the redundancy analysis (RDA) showing significant relationship between metabolic-related factors (response variables) and microbial groups (explanatory variables). First and second ordination axes were plotted, representing 22.6% and 7.9% of the variability in the data set, respectively. *P*-values obtained by Monte Carlo test were reported. For each sample code, the first letter *T* represents transgenic fish, and *C* wild-type controls; the middle letter(s) indicates the diet treatments (*C*: control diet, *HP*: high protein diet, *HC*: high carbohydrate diet, *HL*: high lipid diet), and the last numbers represent replicated samples. GLU represents glucose, AST aspartate aminotransferase, and HDL high-density lipoprotein.

Canonical ordination of redundancy analysis (RDA) was employed to explore the potential relationships between gut microbiota and host’s metabolism. In this analysis, the host metabolic factors resulting from biochemical analysis were used as response variables and gut microbial groups, which were measured by high-throughput sequencing of 16S rRNA genes, as explanatory variables. As shown in Figure 5, three major phyla (Proteobacteria, Bacteroidetes, and Firmicutes) display significant relationships (Monte Carlo test *p*<0.05) with the host metabolic factors, and 96.7% of the response-explanatory variable relation can be significantly explained by the first two axes (*p*<0.05) (Table 2). Moreover, the Bacteroidetes and Firmicutes display a close correlation in predicting the host’s metabolism, which is best evidenced by a small angle between these two variables. On the other hand, Proteobacteria is not correlated with Bacteroidetes and Firmicutes in explaining the host’s metabolism, as large angles exist between Firmicutes and Proteobacteria as well as between Bacteroidetes and Proteobacteria.

**Table 2:** Summary statistics of redundancy analysis (RDA) showing the relationships between gut microbiota and host metabolism by canonical axes with associated *p* values from Monte Carlo test.

Axes	1	2	3	4
Eigenvalues	0.226	0.079	0.010	0.279
Response-explanatory variable correlation	0.700	0.558	0.337	0.000
Cumulative percentage variance				
of response data	22.6	30.5	31.5	59.4
of response-explanatory variables relation	71.8	96.7	100.0	0.0
Significance test of the first canonical axis (*p* value)	0.012			
Significance test of all canonical axes (*p* value)	0.006			

### Transgenic Fish Displays Increased Carbohydrate but Decreased Lipid Metabolisms

**Figure 6:**
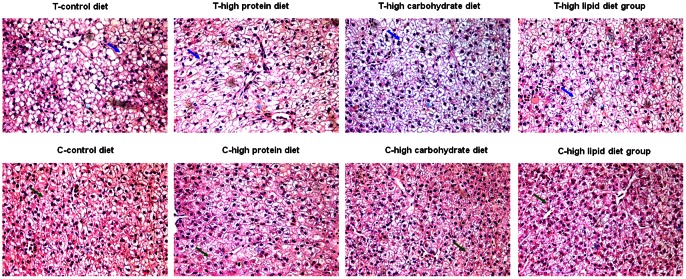
Histological analyses of liver tissues. Liver tissues from 4 different diet treatments were formaldehyde-fixed, followed by staining with Hematoxylin and eosin. The results (75×) from transgenic fish are shown in the upper panels, and wild-type controls with corresponding diet treatments in the lower panels. Arrows in upper panels indicate lipid droplet (blue) and those in lower panels (green) show glycogen deposits.

Physiological and biochemical analyses were performed to study the metabolic differences between transgenic fish and wild-type controls. The results (Table 3) indicated that transgenic fish had significantly lower concentration of glucose (GLU) than that of wild-type controls across all diet treatments and that high carbohydrate diet significantly increased the amount of gut amylase in transgenic fish, suggesting an increased carbohydrate digestion. On the contrary, the lipid-related metabolic parameters such as serum alanine aminotransferase (ALT), aspartate aminotransferase (AST), and triglyceride (TG), which reflect a certain degree of liver dysfunction and represent lipid deposits in the liver, were generally higher in transgenic fish than those of wild-type controls (*p*<0.05), indicating lipid metabolism in transgenic fish was partially disturbed. However, no significant difference was observed for protein metabolism between transgenic fish and wild-type controls. This was evidenced by similar levels of trypsin across 4 types of diets and of all serum indices (except GLU). Supporting these findings, histological analyses of liver tissues revealed that samples from transgenic fish had enlarged cells with increased amount of lipid droplets, while liver tissues from wild-type fish harbored more glycogen deposits (Figure 6).

**Table 3:** Comparison of metabolic differences between transgenic and wild-type fish.

	Control diet	High protein	High carbohydrate	High lipid
Whole-fish lipid (wet weight)	0.04 (T>C)	0.02 (T>C)	NS	0.01 (T>C)
Whole-fish protein (wet weight)	NS	NS	0.01 (T<C)	NS
Whole-fish ash (wet weight)	NS	NS	NS	NS
Whole-fish water	0.02 (T<C)	0.03 (T<C)	NS	0.02 (T<C)
Whole-fish energy	NS	NS	NS	NS
Muscle lipid (wet weight)	NS	0.04 (T<C)	NS	NS
Muscle protein (wet weight)	0.00 (T<C)	NS	NS	NS
Muscle ash (wet weight)	NS	0.01 (T<C)	NS	NS
Muscle water	0.01 (T>C)	0.02 (T>C)	0.04 (T>C)	0.02 (T>C)
Muscle energy	NS	NS	NS	NS
Liver lipid (wet weight)	0.04 (T>C)	0.01 (T>C)	NS	0.01 (T>C)
Amylase	NS	NS	0.04 (T>C)	NS
Trypsin	NS	NS	NS	NS
Serum glucose	0.02 (T<C)	0.01 (T<C)	0.02 (T<C)	0.02 (T<C)
Serum cholesterol	NS	NS	NS	NS
Serum triglyceride	NS	NS	0.01 (T>C)	NS
Serum alanine aminotransferase	0.00 (T>C)	NS	0.00 (T>C)	0.00 (T>C)
Serum aspartate aminotransferase	0.03 (T>C)	NS	0.00 (T>C)	0.04 (T>C)
Serum high-density lipoprotein	0.01 (T<C)	NS	0.04 (T<C)	0.03 (T<C)
Serum low-density lipoprotein	0.04 (T<C)	NS	0.00 (T<C)	NS

The numbers represent the significant *p* values, and the subsequent parenthesis show in which directions the differences are. NS indicates not significant. The letter *T* in parenthesis represents transgenic fish, and *C* wild-type control.

## Discussion

Gut microbiota is a complex community of microorganisms that colonizes the gastrointestinal tract of animals. A growing number of studies have shown gut microbiotas, which are involved in energy harvest and storage as well as in a variety of metabolic functions such as fermenting and absorbing undigested carbohydrates [41], are especially important for host’s metabolism [8]–[11]. Changes in gut microbiota composition are associated with many diseases such as celiac disease [42], austim [43], and obesity [15], [17]. The latter is most likely associated with the changes of two microbiota divisions Bacteroidetes and Firmicutes.

The present study on the difference of gut microbiota composition between wild-type and fast-growing transgenic fish allows us to: i) investigate the associations between gut microbes and the metabolism of fish and ii) address the question whether the change of microbiota Bacteroidetes over Firmicutes is unique to obesity in human. The comparison of gut microbiota between transgenic and wild-type fish provides an analogy but different comparison to obese and lean people in terms of body weight and size. Unlike obesity in humans the large body size of transgenic fish displays a proportional body growth, which has been demonstrated by previous reports [22], [23] and was also confirmed in this study (Figure S2). Different factors might contribute to the fast-growing nature of transgenic fish such as expression of the integrated growth hormone gene [44], increased food intake [45], improved feed efficiency [46], and altered fish behaviors [47], [48].

A core microbiota comprised of Proteobacteria, Fusobacteria, Bacteroidetes, and Firmicutes was identified herein. Proteobacteria accounted for the largest proportion of gut microbiota, which is in agreement with the results from other fish [49], [50]. We, however, observed significant differences of gut microbiota composition between transgenic fish and wild-type controls. It is also very important to note that the largest differences between transgenic fish and wild-type controls are during the early developmental stages (during 2-month and 5-month), for which both Sørensen and *S*_RC_ indices display significant differences. The results were further confirmed by high-throughput sequencing on bacterial 16S rRNA genes. These findings suggest that gut microbiota composition is most likely filtered to be different between transgenic fish and controls at particular developmental stages.

One purpose of this study is to explore the association between gut microbes and fish metabolism, in turn, their growth. Transgenic fish have a significantly faster growth rate, when compared to wild-type controls. This is true for fish reared in both nature (ponds) and laboratory conditions (tanks). This fast-growing characteristic is correlated with gross energy intake and growth energy, both of which are significantly more than those from wild-type fish (Figure S2). This leads to an interesting question as to whether gross energy intake for fast-growing transgenic fish is linked to gut microbiotas. We found 3 out of the 4 core microbiotas were associated with host’s metabolism. This has been illustrated by canonical ordination of redundancy analysis, from which three microbiotas Proteobacteria, Bacteroidetes, and Firmicutes present significant relationships (Monte Carlo test *p*<0.05) with host metabolic characteristics. We also noticed that Bacteroidetes displayed a close correlation with Firmicutes in predicting host metabolism. Therefore, other than the important role played by the dominating phylum Proteobacteria, both Bacteroidetes and Firmicutes could have some impact on fish metabolism. Further analyses are needed to reveal whether the large body size of transgenic fish is the consequence of the gut microbiota change or the cause for the change of gut microbiota.

These findings not only indicate microbiotas play very important roles in host metabolism but also suggest a close connection between gross energy intake and fast-growing feature of transgenic fish. The large gross energy intake in transgenic fish might come from increased carbohydrate metabolisms, which was demonstrated by both biochemical and histological analyses. While transgenic fish had significantly lower concentrations of glucose than those of wild-type fish, high carbohydrate diet significantly increased the amount of gut amylase. Histological analysis of liver sections also revealed liver tissues from wild-type fish harbored more glycogen deposits. These results indicated transgenic fish could take the advantage of carbohydrate diets for the fast growth. This may be partially attributed from the high efficiency of polysaccharide fermentation by Firmicutes [15], [51], [52], as more Firmicutes were detected in the gut of transgenic individuals.

Another important question is whether the increase of Firmicutes at the expense of Bacteroidetes is unique to mammalian obesity. Although previous studies have shown that the increased proportion of Firmicutes have a direct connection with the development of obesity [14]–[18], other studies have differing conclusions [19]–[21]. The present study therefore could shed light on these contradicted findings, as the gut microbiota between transgenic fish and wild-type control provide an analogy but different comparison to obese and lean mammalian individual in terms of body weight and size. Similar to the results from mammals, both RTQ-PCR quantification of 16S rDNA (V3 region) copies and high-throughput sequencing on 16S rDNA (V1-V3 regions) reveal lower Bacteroidetes:Firmicutes ratio in transgenic fish. These results demonstrate that the change of microbiota Bacteroidetes over Firmicutes is also true to the fast-growing transgenic fish. Since transgenic fish has a proportional body growth which is unlike obese individual in mammals, the finding therefore suggests that the link between obesity and microbiota is likely more complex than the simple Bacteroidetes:Firmicutes ratio change [17].

Further evidence comes from similar mechanism for excessive energy harvest in both transgenic fish and obese mammals, for which gut microbiota affects body weight by increasing energy harvest from dietary fibers. Metagenomic and biochemical analyses have revealed that mouse gut microbiota is enriched with bacterial genes capable of fermenting dietary fibers [52]. The notion of changing energy harvest by gut microbiota has also been explored in human by Jumpertz et al. [21], who tested whether microbiota in lean and obese individuals were correlated with the efficiency of dietary energy harvest. They found that the changes of gut microbiota were directly correlated with stool energy loss in lean individuals and that a 20% increase in Firmicutes and a corresponding decrease in Bacteroidetes were associated with an increased energy harvest. Therefore, excessive calories from fiber by microbiota metabolism could be one of the important factors contributing to obese state, which is in good agreement with gut microbes affecting the metabolism of vertebrate fish from this study. These results indicate that the relative abundance of Firmicutes over Bacteroidetes could be one of the factors contributing to the fast growth of transgenic common carp, even though they account for only a small proportion of the total gut microbiota.

## Supporting Information

Figure S1 **Venn diagrams displaying similarity and difference for all phyla and 4 major bacterial phyla between transgenic fish and wild-type controls.** The number of shared members is listed in the middle, the number of members unique to transgenic fish is shown on the left, and that unique to wild-type controls is indicated on the right. *T* represents transgenic fish and *C* wild-type controls.
(TIF)

Figure S2 **Comparison of body weight and energy intake between the fast-growing transgenic fish (** ***T*** **) and wild-type controls (** ***C*** **).** (a) Body weight comparison between transgenic fish and wild-type controls reared in ponds. (b) Body weight comparison between transgenic fish and wild-type controls raised in laboratory tanks. (c) Comparison of condition factor (100 × weight/length^3^) between transgenic fish and wild-type controls reared in ponds. (d) Comparison of condition factor between transgenic fish and wild-type controls raised in laboratory tanks. (e) Gross energy intake comparison between transgenic fish and wild-type controls raised in laboratory tanks. (f) Growth energy comparison between transgenic fish and wild-type controls raised in laboratory tanks. *I_E_* and *G_E_* represent gross energy intake and growth energy, respectively. *W_i_* stands for the initial value of weight and *W_f_* the final weight. Asterisks indicate significant differences for the comparisons obtained from two-tailed Student’s *t*-test (* stands for *p*<0.05 and ** *p*<0.005).
(TIF)

Table S1 Formulation and chemical composition of the experimental diet.
(DOC)

Table S2 Oligonucleotide sequences of all PCR primers used in the study.
(DOC)

## References

[1] Ley RE, Hamady M, Lozupone C, Turnbaugh PJ, Ramey RR, et al (2008) Evolution of mammals and their gut microbes. Science 320: 1647–1651. 18497261 10.1126/science.1155725 PMC2649005

[2] Li X, Yu Y, Feng W, Yan Q, Gong Y (2012) Host species as a strong determinant of the intestinal microbiota of fish larvae. J Microbiol 50: 29–37. 22367934 10.1007/s12275-012-1340-1

[3] Rawls JF, Mahowald MA, Ley RE, Gordon JI (2006) Reciprocal gut microbiota transplants from zebrafish and mice to germ-free recipients reveal host habitat selection. Cell 127: 423–433. 17055441 10.1016/j.cell.2006.08.043 PMC4839475

[4] Yan Q, van der Gast CJ, Yu Y (2012) Bacterial community assembly and turnover within the intestines of developing zebrafish. PLoS ONE 7: e30603. 22276219 10.1371/journal.pone.0030603 PMC3261916

[5] Bäckhed F, Manchester JK, Semenkovich CF, Gordon JI (2007) Mechanisms underlying the resistance to diet-induced obesity in germ-free mice. Proc Natl Acad Sci U S A 104: 979–984. 17210919 10.1073/pnas.0605374104 PMC1764762

[6] O’Hara AM, Shanahan F (2006) The gut flora as a forgotten organ. EMBO Rep 7: 688–693. 16819463 10.1038/sj.embor.7400731 PMC1500832

[7] Qin J, Li R, Raes J, Arumugam M, Burgdorf KS, et al (2010) A human gut microbial gene catalogue established by metagenomic sequencing. Nature 464: 59–65. 20203603 10.1038/nature08821 PMC3779803

[8] Bäckhed F (2011) Programming of host metabolism by the gut microbiota. Ann Nutr Metab 58: 44–52. 21846980 10.1159/000328042

[9] Nicholson JK, Holmes E, Wilson ID (2005) Gut microorganisms, mammalian metabolism and personalized health care. Nat Rev Microbiol 3: 431–438. 15821725 10.1038/nrmicro1152

[10] Stevens CE, Hume ID (1998) Contributions of microbes in vertebrate gastrointestinal tract to production and conservation of nutrients. Physiol Rev 78: 393–427. 9562034 10.1152/physrev.1998.78.2.393

[11] Velagapudi VR, Hezaveh R, Reigstad CS, Gopalacharyulu P, Yetukuri L, et al (2010) The gut microbiota modulates host energy and lipid metabolism in mice. J Lipid Res 51: 1101–1112. 20040631 10.1194/jlr.M002774 PMC2853437

[12] Friedman JM (2004) Modern science versus the stigma of obesity. Nat Med 10: 563–569. 15170194 10.1038/nm0604-563

[13] Musso G, Gambino R, Cassader M (2011) Interactions between gut microbiota and host metabolism predisposing to obesity and diabetes. Annu Rev Med 62: 361–380. 21226616 10.1146/annurev-med-012510-175505

[14] Turnbaugh PJ, Bäckhed F, Fulton L, Gordon JI (2008) Diet-induced obesity is linked to marked but reversible alterations in the mouse distal gut microbiome. Cell Host Microbe 3: 213–223. 18407065 10.1016/j.chom.2008.02.015 PMC3687783

[15] Ley RE, Turnbaugh PJ, Klein S, Gordon JI (2006) Microbial ecology: human gut microbes associated with obesity. Nature 444: 1022–1023. 17183309 10.1038/4441022a

[16] Santacruz A, Marcos A, Wärnberg J, Marti A, Martin-Matillas M, et al (2009) Interplay between weight loss and gut microbiota composition in overweight adolescents. Obesity 17: 1906–1915. 19390523 10.1038/oby.2009.112

[17] Turnbaugh PJ, Hamady M, Yatsunenko T, Cantarel BL, Duncan A, et al (2009) A core gut microbiome in obese and lean twins. Nature 457: 480–484. 19043404 10.1038/nature07540 PMC2677729

[18] Zhang H, DiBaise JK, Zuccolo A, Kudrna D, Braidotti M, et al (2009) Human gut microbiota in obesity and after gastric bypass. Proc Natl Acad Sci U S A 106: 2365–2370. 19164560 10.1073/pnas.0812600106 PMC2629490

[19] Duncan SH, Lobley GE, Holtrop G, Ince J, Johnstone AM, et al (2008) Human colonic microbiota associated with diet, obesity and weight loss. Int J Obes 32: 1720–1724. 10.1038/ijo.2008.155 18779823

[20] Schwiertz A, Taras D, Schafer K, Beijer S, Bos NA, et al (2010) Microbiota and SCFA in lean and overweight healthy subjects. Obesity 18: 190–195. 19498350 10.1038/oby.2009.167

[21] Jumpertz R, Le DS, Turnbaugh PJ, Trinidad C, Bogardus C, et al (2011) Energy-balance studies reveal associations between gut microbes, caloric load, and nutrient absorption in humans. Am J Clin Nutr 94: 58–65. 21543530 10.3945/ajcn.110.010132 PMC3127503

[22] Zhu Z (1992) Growth hormone gene and the transgenic fish. In: You CB, Chen ZL, editors. Agricultural Biotechnology. Beijing: China Science and Technology Press. 106–116.

[23] Zhu Z (1992) Generation of fast growing transgenic fish: Methods and mechanisms. In: Hew CL, Fletcher GL, editors. Transgenic Fish. Singapore: World Scientific Publishing. 92–119.

[24] Erman A, Veilleux A, Tchernof A, Goodyer CG (2011) Human growth hormone receptor (GHR) expression in obesity: I. GHR mRNA expression in omental and subcutaneous adipose tissues of obese women. Int J Obes 35: 1511–1519. 10.1038/ijo.2011.23 21386804

[25] Berryman DE, List EO, Palmer AJ, Chung MY, Wright-Piekarski J, et al (2010) Two-year body composition analyses of long-lived GHR null mice. J Gerontol A Biol Sci Med Sci 65: 31–40. 19901018 10.1093/gerona/glp175 PMC2796884

[26] Rawls JF, Samuel BS, Gordon JI (2004) Gnotobiotic zebrafish reveal evolutionarily conserved responses to the gut microbiota. Proc Natl Acad Sci U S A 101: 4596–4601. 15070763 10.1073/pnas.0400706101 PMC384792

[27] Li XM, Yu YH, Xie SQ, Yan QY, Chen YH (2011) Effect of chitosan on intestinal bacteria of allogynogenetic crucian carp, *Carassius auratus gibelio*, as depicted by polymerase chain reaction-denaturing gradient gel electrophoresis. J World Aquacult Soc 42: 539–548.

[28] Muyzer G, de Waal EC, Uitterlinden AG (1993) Profiling of complex microbial populations by denaturing gradient gel electrophoresis analysis of polymerase chain reaction-amplified genes coding for 16S rRNA. Appl Environ Microbiol 59: 695–700. 7683183 10.1128/aem.59.3.695-700.1993 PMC202176

[29] Yu Y, Yan Q, Feng W (2008) Spatiotemporal heterogeneity of plankton communities in Lake Donghu, China, as revealed by PCR-denaturing gradient gel electrophoresis and its relation to biotic and abiotic factors. FEMS Microbiol Ecol 63: 328–337. 18205816 10.1111/j.1574-6941.2007.00430.x

[30] Cole JR, Chai B, Farris RJ, Wang Q, Kulam SA, et al (2005) The Ribosomal Database Project (RDP-II): sequences and tools for high-throughput rRNA analysis. Nucleic Acids Res 33: D294–296. 15608200 10.1093/nar/gki038 PMC539992

[31] Tamura K, Dudley J, Nei M, Kumar S (2007) MEGA4: Molecular Evolutionary Genetics Analysis (MEGA) software version 4.0. Mol Biol Evol 24: 1596–1599. 17488738 10.1093/molbev/msm092

[32] Guo X, Xia X, Tang R, Zhou J, Zhao H, et al (2008) Development of a real-time PCR method for Firmicutes and Bacteroidetes in faeces and its application to quantify intestinal population of obese and lean pigs. Lett Appl Microbiol 47: 367–373. 19146523 10.1111/j.1472-765X.2008.02408.x

[33] Schloss PD (2010) The effects of alignment quality, distance calculation method, sequence filtering, and region on the analysis of 16S rRNA gene-based studies. PLoS Comput Biol 6: e1000844. 20628621 10.1371/journal.pcbi.1000844 PMC2900292

[34] Caporaso JG, Lauber CL, Walters WA, Berg-Lyons D, Lozupone CA, et al (2011) Global patterns of 16S rRNA diversity at a depth of millions of sequences per sample. Proc Natl Acad Sci U S A 108: 4516–4522. 20534432 10.1073/pnas.1000080107 PMC3063599

[35] Caporaso JG, Kuczynski J, Stombaugh J, Bittinger K, Bushman FD, et al (2010) QIIME allows analysis of high-throughput community sequencing data. Nat Methods 7: 335–336. 20383131 10.1038/nmeth.f.303 PMC3156573

[36] Hamady M, Lozupone C, Knight R (2010) Fast UniFrac: facilitating high-throughput phylogenetic analyses of microbial communities including analysis of pyrosequencing and PhyloChip data. ISME J 4: 17–27. 19710709 10.1038/ismej.2009.97 PMC2797552

[37] Raup DM, Crick RE (1979) Measurement of faunal similarity in paleontology. Journal Paleontol 53: 1213–1227.

[38] Rowan AK, Snape JR, Fearnside D, Barer MR, Curtis TP, et al (2003) Composition and diversity of ammonia-oxidising bacterial communities in wastewater treatment reactors of different design treating identical wastewater. FEMS Microbiol Ecol 43: 195–206. 19719680 10.1111/j.1574-6941.2003.tb01059.x

[39] Costello EK, Gordon JI, Secor SM, Knight R (2010) Postprandial remodeling of the gut microbiota in Burmese pythons. ISME J 4: 1375–1385. 20520652 10.1038/ismej.2010.71 PMC3923499

[40] Semova I, Carten JD, Stombaugh J, Mackey LC, Knight R, et al (2012) Microbiota regulate intestinal absorption and metabolism of fatty acids in the zebrafish. Cell Host & Microbe 12: 277–288. 22980325 10.1016/j.chom.2012.08.003 PMC3517662

[41] Gill SR, Pop M, Deboy RT, Eckburg PB, Turnbaugh PJ, et al (2006) Metagenomic analysis of the human distal gut microbiome. Science 312: 1355–1359. 16741115 10.1126/science.1124234 PMC3027896

[42] Elinav E, Strowig T, Kau AL, Henao-Mejia J, Thaiss CA, et al (2011) NLRP6 inflammasome regulates colonic microbial ecology and risk for colitis. Cell 145: 745–757. 21565393 10.1016/j.cell.2011.04.022 PMC3140910

[43] Robinson CJ, Bohannan BJ, Young VB (2010) From structure to function: the ecology of host-associated microbial communities. Microbiol Mol Biol Rev 74: 453–476. 20805407 10.1128/MMBR.00014-10 PMC2937523

[44] Wang YP, Hu W, Wu G, Sun YH, Chen SP, et al (2001) Genetic analysis of “all-fish” growth hormone gene transferred carp (*Cyprinus carpio* L.) and its F1 generation. Chin Sci Bull 46: 1175–1179.

[45] Fu C, Li D, Hu W, Wang Y, Zhu Z (2007) Growth and energy budget of F2 ‘all-fish’ growth hormone gene transgenic common carp. J Fish Biol 70: 347–361.

[46] Guan B, Hu W, Zhang TL, Wang YP, Zhu ZY (2008) Metabolism traits of ‘all-fish’ growth hormone transgenic common carp. Aquaculture 284: 217–223.

[47] Duan M, Zhang TL, Hu W, Li ZJ, Sundström LF, et al (2011) Behavioral alterations in GH transgenic common carp may explain enhanced competitive feeding ability. Aquaculture 317: 175–181.

[48] Li DL, Fu CZ, Hu W, Zhong S, Wang YP, et al (2007) Rapid growth cost in “all-fish” growth hormone gene trainsgenic carp: reduced critical swimming speed. Chin Sci Bull 52: 1501–1506.

[49] Roeselers G, Mittge EK, Stephens WZ, Parichy DM, Cavanaugh CM, et al (2011) Evidence for a core gut microbiota in the zebrafish. ISME J 5: 1595–1608. 21472014 10.1038/ismej.2011.38 PMC3176511

[50] Wu S, Wang G, Angert ER, Wang W, Li W, et al (2012) Composition, diversity, and origin of the bacterial community in grass carp intestine. PLoS ONE 7: e30440. 22363439 10.1371/journal.pone.0030440 PMC3282688

[51] Bäckhed F, Ding H, Wang T, Hooper LV, Koh GY, et al (2004) The gut microbiota as an environmental factor that regulates fat storage. Proc Natl Acad Sci U S A 101: 15718–15723. 15505215 10.1073/pnas.0407076101 PMC524219

[52] Turnbaugh PJ, Ley RE, Mahowald MA, Magrini V, Mardis ER, et al (2006) An obesity-associated gut microbiome with increased capacity for energy harvest. Nature 444: 1027–1031. 17183312 10.1038/nature05414

