# Supplementary figures and images for: Gut Microbiota Contributes to the Growth of Fast-Growing Transgenic Common Carp (Cyprinus carpio L.)
Source: PLoS One. 2013 May 31;8(5):e64577. doi: 10.1371/journal.pone.0064577 (PMC3669304; doi:10.1371/journal.pone.0064577)

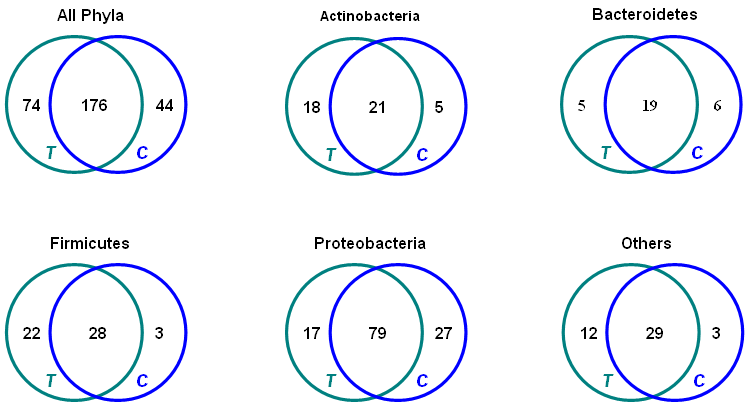

Supplement: Figure S1 — Venn diagrams displaying similarity and difference for all phyla and 4 major bacterial phyla between transgenic fish and wild-type controls. The number of shared members is listed in the middle, the number of members unique to transgenic fish is shown on the left, and that unique to wild-type controls is indicated on the right. T represents transgenic fish and C wild-type controls. (TIF) [file pone.0064577.s001.tif]

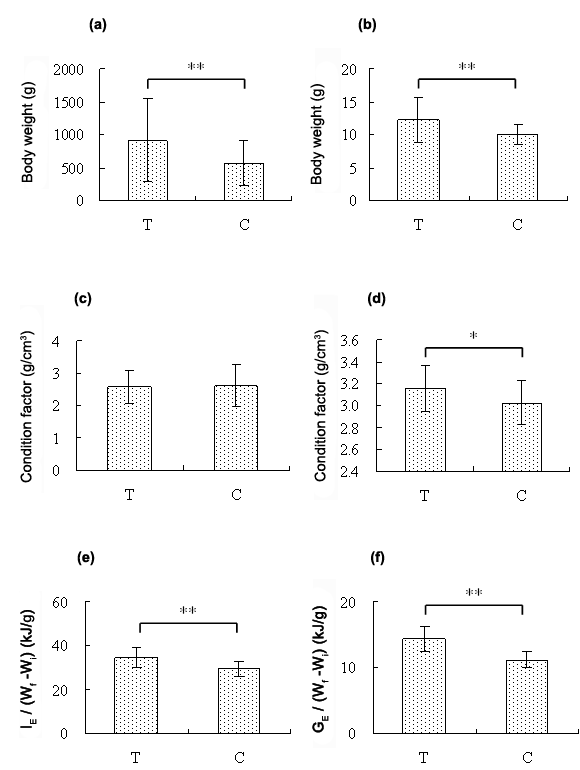

Supplement: Figure S2 — Comparison of body weight and energy intake between the fast-growing transgenic fish ( T ) and wild-type controls ( C ). (a) Body weight comparison between transgenic fish and wild-type controls reared in ponds. (b) Body weight comparison between transgenic fish and wild-type controls raised in laboratory tanks. (c) Comparison of condition factor (100 × weight/length3) between transgenic fish and wild-type controls reared in ponds. (d) Comparison of condition factor between transgenic fish and wild-type controls raised in laboratory tanks. (e) Gross energy intake comparison between transgenic fish and wild-type controls raised in laboratory tanks. (f) Growth energy comparison between transgenic fish and wild-type controls raised in laboratory tanks. IE and GE represent gross energy intake and growth energy, respectively. Wi stands for the initial value of weight and Wf the final weight. Asterisks indicate significant differences for the comparisons obtained from two-tailed Student’s t-test (* stands for p<0.05 and ** p<0.005). (TIF) [file pone.0064577.s002.tif]
