# Supplementary material for: Gut Microbiota Contributes to the Growth of Fast-Growing Transgenic Common Carp (Cyprinus carpio L.)
Source: PLoS One. 2013 May 31;8(5):e64577. doi: 10.1371/journal.pone.0064577 (PMC3669304; doi:10.1371/journal.pone.0064577)
Supplement: Table S1 — Formulation and chemical composition of the experimental diet. (DOC) [file pone.0064577.s003.doc]

**Table S1.** Formulation and chemical composition of the experimental diet.

|  | *Control diet* | *High protein* | *High carbohydrate* | *High*  *lipid* |
| --- | --- | --- | --- | --- |
| *Ingredients (% dry matter)* |  |  |  |  |
| Fishmeal | 32.2 | 41.7 | 29.2 | 29.2 |
| Soybean | 20.0 | 25.8 | 18.1 | 18.1 |
| Starch | 31.8 | 14.5 | 43.0 | 20.8 |
| Fish oil | 4.25 | 3.3 | 4.1 | 12.7 |
| Cellulose | 6.5 | 9.5 | 0.4 | 14.0 |
| Vitamin premix | 2.0 | 2.0 | 2.0 | 2.0 |
| Choline chloride | 0.1 | 0.1 | 0.1 | 0.1 |
| Yi_2_O_3_ | 0.1 | 0.1 | 0.1 | 0.1 |
| Carboxymethyl cellulose sodium (CMC) | 3.0 | 3.0 | 3.0 | 3.0 |
|  |  |  |  |  |
| *Chemical composition (% dry matter)* |  |  |  |  |
| Dry matter | 99.04 | 99.05 | 99.08 | 99.08 |
| Crude protein | 33.60 | 42.10 | 30.06 | 29.60 |
| Crude lipid | 6.26 | 6.34 | 6.48 | 12.92 |
| Ash | 9.50 | 11.75 | 8.55 | 8.44 |
| Gross energy (kJ/g) | 16.41 | 17.02 | 17.02 | 17.84 |
