# Supplementary material for: Gut Microbiota Contributes to the Growth of Fast-Growing Transgenic Common Carp (Cyprinus carpio L.)
Source: PLoS One. 2013 May 31;8(5):e64577. doi: 10.1371/journal.pone.0064577 (PMC3669304; doi:10.1371/journal.pone.0064577)
Supplement: Table S2 — Oligonucleotide sequences of all PCR primers used in the study. (DOC) [file pone.0064577.s004.doc]

**Table S2.** Oligonucleotide sequences of all PCR primers used in the study.

| *Assay* | *Primer code* | *Oligonucleotide sequence (5'-3')* |
| --- | --- | --- |
| DGGE | 357F-GC* | CCTACGGGAGGCAGCAG |
|  | 518R | ATTACCGCGGCTGCTGG |
| Q-PCR (Firmicutes) | Firm934F | GGAGYATGTGGTTTAATTCGAAGCA |
|  | Firm1060R | AGCTGACGACAACCATGCAC |
| Q-PCR (Bacteroidetes) | Bact934F | GGARCATGTGGTTTAATTCGATGAT |
|  | Bact1060R | AGCTGACGACAACCATGCAG |
| Q-PCR (All bacteria) | Eub338F | ACTCCTACGGGAGGCAGCAG |
|  | Eub518R | ATTACCGCGGCTGCTGG |
| Pyrosequencing | 27F | AGAGTTTGATCCTGGCTCAG |
|  | 534R | ATTACCGCGGCTGCTGG |

* With a GC clamp (CGCCCGCCGCGCCCCGCGCCCGGCCCGCCGCCCCCGCCCC) at the 5’ end.
